# *DUSP1* Gene Polymorphisms Are Associated with Obesity-Related Metabolic Complications among Severely Obese Patients and Impact on Gene Methylation and Expression

**DOI:** 10.1155/2013/609748

**Published:** 2013-08-06

**Authors:** F. Guénard, L. Bouchard, A. Tchernof, Y. Deshaies, F. S. Hould, S. Lebel, P. Marceau, L. Pérusse, M. C. Vohl

**Affiliations:** ^1^Institute of Nutrition and Functional Foods (INAF), Laval University, Québec, QC, Canada G1V 0A6; ^2^Department of Food Science and Nutrition, Laval University, Québec, QC, Canada G1V 0A6; ^3^Endocrinology and Nephrology, CHU de Québec Research Center, Québec, QC, Canada G1V 4G2; ^4^Department of Biochemistry, Université de Sherbrooke, ECOGENE-21, Chicoutimi Hospital, Saguenay, Canada G7H 7P2; ^5^Department of Medicine, Laval University, Québec, QC, Canada G1V 0A6; ^6^Québec Heart and Lung Institute, Québec, QC, Canada G1V 4G5; ^7^Department of Surgery, Laval University, Québec, QC, Canada G1V 0A6; ^8^Department of Kinesiology, Laval University, Québec, QC, Canada G1V 0A6

## Abstract

The *DUSP1* gene encodes a member of the dual-specificity phosphatase family previously identified as being differentially expressed in visceral adipose tissue (VAT) of severely obese men with versus without the metabolic syndrome. *Objective.* To test the association between *DUSP1* polymorphisms, obesity-related metabolic complications, gene methylation, and expression levels in VAT. *Methods.* The *DUSP1* locus and promoter region were sequenced in 25 individuals. SNPs were tested for association with obesity-related complications in a cohort of more than 1900 severely obese individuals. The impact of SNPs on methylation levels of 36 CpG sites and correlations between DNA methylation and gene expression levels in VAT were computed in a subset of 14 samples. *Results.* Heterozygotes for rs881150 had lower HDL-cholesterol levels (HDL-C; *P* = 0.01), and homozygotes for the minor allele of rs13184134 and rs7702178 had increased fasting glucose levels (*P* = 0.04 and 0.01, resp.). rs881150 was associated with methylation levels of CpG sites located ~1250 bp upstream the transcription start site. Methylation levels of 4 CpG sites were inversely correlated with *DUSP1* gene expression. *Conclusion.* These results suggest that *DUSP1* polymorphisms modulate plasma glucose and HDL-C levels in obese patients possibly through alterations of DNA methylation and gene expression levels.

## 1. Introduction

Obesity in adults and in adolescents significantly increases the risk for several chronic diseases including cardiovascular diseases (CVD) and type 2 diabetes (T2D) [1, 2]. Excess accumulation of fat inside the abdominal cavity (visceral adipose tissue: VAT) is associated with altered insulin sensitivity, blood pressure (BP), and plasma lipid profile [3, 4]. Clustering of CVD risk factors including accumulation of abdominal fat, impaired glucose tolerance, dyslipidemia, and hypertension defines the metabolic syndrome (MetS) [5]. Interindividual variability in obesity-related complications is observed. Not all obese individuals develop the MetS, and as much as 20% of the obese population could have normal metabolic parameters even in the presence of extensive adipose tissue accumulation [6]. Our knowledge of the factors involved in the etiology of obesity-related metabolic disturbances, either alone or clustered under the MetS designation, remains largely incomplete, with evidence pointing to a potential implication of immunity and inflammatory processes as well as oxidative stress [7, 8]. There is also strong evidence for genetic components in the development of these perturbations [9]. In a previous transcriptomic study aimed at the identification of genes predisposing to MetS in established obesity, we have identified 489 differentially expressed genes in VAT of nondiabetic obese men with the MetS (MetS+) in comparison with obese men without the MetS (MetS−) [10]. One of the 489 genes differentially expressed was the *DUSP1* gene.

DUSP1, also known as MKP1, is a member of the dual-specificity phosphatase family involved in the regulation of the innate immune function [11]. Following p53-mediated transcription, DUSP1 targets p38, MAPKs, and JNKs which are important players in the expression of inflammatory mediators [12, 13]. In addition, it mediates the cellular response to oxidative stress leading to apoptosis [14]. Differences in *DUSP1* gene expression have been reported in various disorders. *DUSP1* overexpression has been reported in breast, gastric, and lung cancers [15–17]. In contrast, downregulation was significantly correlated to increased tumor aggressiveness and reduced patient survival in hepatocellular carcinoma (HCC), urothelial, prostate, and liver cancers [18–21]. Downregulation of *DUSP1* with concomitant promoter hypermethylation and loss of heterozygosity at the *DUSP1* locus was reported in HCC [18], and epigenetic regulation of *DUSP1* expression was suggested in prostate cancer [19].

Based on the functions of DUSP1 in innate immune function, inflammation, and apoptosis and its implication in various disorders and differential expression we have shown in VAT of MetS+ versus MetS− obese individuals [10], we postulated that *DUSP1* gene variations may contribute to partially explain interindividual variability observed in obesity-related metabolic complication among obese individuals through the regulation of gene methylation and expression. An extensive molecular analysis of the human *DUSP1* gene was undertaken to test for associations between *DUSP1* polymorphisms and obesity-related metabolic complications in a cohort of severely obese patients undergoing a bariatric surgery. The impact of the SNPs associated with metabolic complications on gene methylation and expression in VAT was thereafter analyzed.

## 2. Material and Methods

### 2.1. Patient Selection

Between June 2000 and July 2012, 1906 severely obese men (*N* = 597) and women (*N* = 1309) have been recruited. These Caucasian patients from the eastern part of the Quebec province, Canada, underwent biliopancreatic diversion with duodenal switch at the Quebec Cardiology and Pulmonology Institute (Quebec City, Quebec, Canada). The study was approved by the Université Laval ethics committee, and all individuals provided written informed consent before their inclusion. The surgical protocol is presented elsewhere [22]. Body weight, height, waist girth, resting BP [systolic (SBP) and diastolic (DBP), blood lipid, and glucose concentrations were measured preoperatively using standardized procedures [23]. Body mass index (BMI) was calculated as weight in kilograms divided by height in meters squared. The presence of MetS was determined using the National Cholesterol Education Program Adult Treatment Panel III (NCEP-ATPIII) guidelines when an individual fulfilled three or more criteria [5].

### 2.2. Gene Expression Analysis

In a previous study [10], a subset of 14 obese men (BMI > 40 kg/m^2^) not taking any medication to treat MetS features were selected from our cohort for gene expression profiling using oligonucleotide microarrays. Seven of them were affected by the MetS (MetS+ group) and were matched for age, BMI, and smoking status to obese individuals without MetS (MetS− group, *N* = 7). Characteristics of these individuals stratified by MetS group as well as procedures for expression profiling are available elsewhere [10]. Following data correction and normalization, the *DUSP1* gene was identified as part of a list of 489 genes differentially expressed in VAT between the two groups.

To validate the microarray results, *DUSP1* gene expression was measured by real-time PCR (RT-PCR) (Applied Biosystems Gene Expression Assays; Applied Biosystems, Foster City, CA, USA) in the same 14 adipose tissue samples selected for the original microarray experiment [10] using custom primers overlapping the first two coding exons. These samples were analyzed in triplicate and calibrated to the *GAPDH* housekeeping gene (endogenous control; *GAPDH*: Hs99999905_m1). Relative quantification estimations were performed on an Applied Biosystems 7500 Fast Real-Time PCR System following the manufacturer's recommendations, and the ΔΔCT calculation method was used to evaluate the mean fold expression difference (MFED) between MetS− and MetS+ groups.

### 2.3. Sequencing and Genotyping

Genomic DNA was extracted from the blood buffy coat using the GenElute Blood Genomic DNA kit (Sigma, St. Louis, MO, USA). The promoter region (~1500 bp), exons, and intronic flanking regions of the *DUSP1* gene were first amplified and sequenced with primers designed using human *DUSP1* public sequence (accession number: NC_000005) in a subset of 25 severely obese men (MetS+ = 12; MetS− = 13) including the 14 individuals selected for gene expression profiling. Additional 11 individuals were randomly selected. MetS status was taken into account to include similar proportions of MetS+ and MetS− individuals for *DUSP1* sequencing. The BigDye Terminator 3.1 kit was used for sequencing, and samples were run on an ABI 3730/XL DNA Analyzer automated sequencer (Applied Biosystems). From the SNPs identified by direct sequencing, five tagging single nucleotide polymorphisms (tSNP) (minor allele frequency ≥5%; MAF) were selected using the tagger selection algorithm of the Haploview software [24, 25]. Selected tSNPs were genotyped using validated primers and TaqMan probes (Applied Biosystems). The rs201026723 polymorphism, resulting in amino acid change (p.Met60Ile), was also included in our analysis and genotyped in the whole cohort (1906 obese individuals) using the same method. Genotypes were determined using Applied Biosystems 7500 Fast Real-Time PCR System and analyzed using ABI Prism SDS version 1.2.3 software (Applied Biosystems). The mean genotype call rate for these 6 SNPs was 97.0%.

Validation of genotyping data was conducted for the SNP rs88150 demonstrating overrepresentation of rare homozygote individuals. PCR amplification and direct sequencing of *DUSP1* promoter region were performed on a subset of 91 consecutively recruited individuals from our cohort (4.8% of the whole cohort). Degree of concordance between TaqMan genotyping and sequencing results was calculated.

### 2.4. DNA Methylation Analysis

DNA methylation analysis was conducted on the 14 obese men initially selected from our cohort for gene expression profiling. Genomic DNA was extracted from 200 mg of VAT using the DNeasy Blood & Tissue kit (QIAGEN, Mississauga, Ontario, Canada), as recommended by the manufacturer. Bisulfite conversion was conducted on 1 *μ*g of DNA, and quantitative DNA methylation analysis was carried out at the McGill University and Génome Québec Innovation Centre (Montreal, Canada). Infinium HumanMethylation450 BeadChip (Illumina Inc., San Diego, CA) was processed according to the manufacturer's instructions. The BeadChip interrogates more than 485000 methylation sites at single-nucleotide resolution. Methylation data was visualized and analyzed using the GenomeStudio software version 2011.1 (Illumina Inc.) and the methylation module. Methylation levels (beta values; *β*) were estimated as the ratio of signal intensity of the methylated alleles to the sum of methylated and unmethylated intensity signals of the alleles (*β* value = C/(T+C)). Data correction (background subtraction and normalization) was applied using internal control probe pairs. CpG sites with a detection *P* value > 0.05 were removed from analysis. Methylation ratios (*β* values) of CpG sites located within the* DUSP1* locus and promoter region (~2 kb upstream transcription start site (TSS)) were extracted using the GenomeStudio Methylation Module, thus leading to a total of 36 CpG sites analyzed in this study.

### 2.5. Statistical Analyses

Phenotypic data (untransformed and unadjusted values) were reported as mean ± SD. Gene expression microarray analysis was conducted using unpaired Student's *t*-test to identify differentially expressed genes between MetS+ and MetS− groups. Hardy-Weinberg equilibrium (HWE), linkage disequilibrium (*r* ^2^), and selection of tSNPs (minor allele frequency; MAF ≥ 5%) were conducted using the Haploview software [24] and the Tagger selection algorithm [25]. After genotyping, homozygotes for the minor allele of the SNP rs201026723 were merged to heterozygotes due to genotype frequency below 5%. Phenotypic differences between genotype groups were tested for the whole cohort (1906 individuals) using analysis of variance (general linear model, type III sum of squares) and adjusted for the effects of age, sex, and BMI. Transformations were applied to nonnormally distributed variables to meet the criteria for normality (inverse transformed: triglycerides (TG) and HDL-cholesterol (HDL-C); log 10 transformed: fasting glucose and total-C (total cholesterol)/HDL-C ratio). When a significant SNP effect was identified, all pairwise comparisons among genotype groups were performed using LS-means and Student's *t*-tests. Pairwise Pearson correlations between methylation and expression levels and between microarray and RT-PCR data were computed. Statistical significance was defined as *P* ≤ 0.05. *P* values presented are for transformed and adjusted values when appropriate. Statistical analyses were performed using SAS software version 9.2 (SAS Institute, Cary, NC, USA).

### 2.6. Bioinformatics Analyses

The potential functional impact of CVD risk factor-associated SNPs was assessed using two different programs. The MatInspector program was used to analyze the impact of promoter SNPs on transcription factors binding sites [26, 27] while intronic SNPs were evaluated using the NNSPLICE 0.9 programs [28]. All tests were run under default threshold values.

## 3. Results

### 3.1. Cohort Description

The present study included 1906 severely obese individuals (597 men and 1309 women) undergoing bariatric surgery who were consecutively recruited. From these individuals, 1899 were successfully classified either as MetS+ (*n* = 1549) or MetS− (*n* = 350 or 18.4%). As shown in Table 1, this cohort of middle-aged, severely obese individuals demonstrates high mean fasting plasma glucose and TG levels, as well as elevated SBP according to MetS criteria defined by the NCEP-ATPIII [5]. Further stratification according to MetS status is also provided in Table 1.

### 3.2. Gene Expression Microarray Results and Validation

As previously reported, microarray analysis of 14 obese men (MetS+, *n* = 7; MetS−, *n* = 7) revealed 489 differentially expressed genes between MetS+ and MetS− groups [10]. Although not listed at the time, the* DUSP1* gene was significantly underexpressed (−3.77-fold; *P* = 0.02) in VAT of MetS+ versus MetS− obese men. Validation of microarray data for the *DUSP1* gene was conducted in the same subset of individuals using RT-PCR. According to RT-PCR results, the *DUSP1* gene was found to be underexpressed in VAT of MetS+ obese men (MFED = −6.02; *P* = 0.04). *DUSP1* gene expression levels obtained by RT-PCR were highly correlated to those obtained by microarrays (*r* = 0.984; *P* < 0.0001). More details on RT-PCR and microarray results are available in Supplementary Table 1 available online at http://dx.doi.org/10.1155/2013/609748.

### 3.3. Identification *DUSP1* SNPs and Selection of tSNPs

Promoter region, exons, and intronic flanking sequences of the *DUSP1* gene were first analyzed in a subset of 25 severely obese individuals and led to the identification of 16 gene variations (Table 2 and Supplementary Figure 1). From these SNPs, 12 were reported in the SNP database (dbSNP build 137) and 7 displayed a MAF ≥ 5%. Common SNPs (MAF ≥ 5%) were found in individuals from MetS− and MetS+ groups. Rare SNPs (MAF < 5%) were mostly found in MetS− individuals with c. 648G>T being identified in a MetS+ individual. Among the 16 SNPs identified, 8 were located in promoter and 3 were intronic. The remaining 5 were exonic polymorphisms, 4 located in the first exon and the other located in exon 3. Three of the exonic SNPs resulted in amino acid changes (p.Asn36His; p.Arg53Leu; p.Met60Ile). Identification of tSNPs was carried out from the 7 SNPs with MAF ≥ 5%. Five SNPs were selected and allowed us to cover 85% of the sequence-derived genetic variability of the common polymorphisms (MAF ≥ 5%) at the *DUSP1* locus. These 5 tSNPs (rs13184134, rs322382, rs881150, rs28372789, and rs7702178) and the rs201026723 (p.Met60Ile) were further genotyped in the whole cohort of 1906 severely obese individuals. The number of genotypes and HWE *P* values obtained are shown in Table 3. One of these SNPs (rs881150, c. −950A>T) displays deviation from HWE with an overrepresentation of rare homozygotes. Validation of TaqMan genotyping results for rs881150 was conducted in a subset of 91 individuals via direct sequencing. All genotypes (100%) were concordant between both methods.

### 3.4. Association of *DUSP1* SNPs with CVD Risk Factors

Associations between fasting plasma glucose, lipid profile BP-related variables (systolic and diastolic blood pressure), and genotype groups were tested. Following adjustment for the effects of age, sex, and BMI, rs881150 was associated with plasma HDL-C levels (*P* = 0.01), with the heterozygotes exhibiting lower levels (Table 4) and minor allele homozygotes having the lower values. SNPs rs13184134 and rs7702178 were associated with fasting glucose levels (*P* = 0.04 and 0.01, resp.), homozygotes for the minor allele of rs13184134 or rs7702178 having increased fasting glucose levels (Table 4).

### 3.5. Potential Impact of SNPs Associated with CVD Risk Factors

Further analyses were conducted for the three SNPs associated with CVD risk factors (rs881150, rs13184134, and rs7702178). Prediction models have been used to assess the potential impact of CVD risk factor-associated SNPs. Analysis of the potential effect of the rs7702178 intronic SNP on splicing revealed that it slightly reduces the strength of an acceptor site located on the reverse strand (0.53 to 0.47), thus unlikely impacting on mRNA splicing for which junction between intron 2 and exon 3 scored 0.88. The MatInspector program was used to analyze the impact of the two CVD risk factor-associated promoter SNPs (rs13184134, rs881150) on binding of transcription factors. Both SNPs demonstrated potential impact (disruption or creation) on transcription factor binding sites based on matrix similarities (Supplementary Table 2). These two promoter variants could thus result in differences in gene expression and contribute to the difference of *DUSP1* expression reported in the current study.

### 3.6. Gene Methylation and Expression Analysis

To explore potential mechanisms underlying the relation between *DUSP1* SNPs and cardiometabolic risk factors, associations between CVD risk factor-associated SNPs (rs881150, rs13184134, and rs7702178) and gene methylation levels were tested. Of the 36 CpG sites analyzed, 25 were located within the promoter region, 8 in exons, and 3 in introns. The list of CpG sites analyzed with corresponding localization is provided in Supplementary Table 3. Significant associations between CpG site methylation levels and genotype groups were identified for 10 CpG sites. A trend towards an association (*P* < 0.10) was also observed for 5 CpG sites. As shown in Table 5, a high proportion of these CpG sites (14/15) was located within the promoter region, with 7 (cg25108022, cg23002268, cg14968860, cg19537645, cg09799633, cg21121138, and cg09493150) being located 1110–1402 bp upstream the TSS (1359-1651 bp from first codon). High level of correlation (mean pairwise correlation; *r* = 0.642) was found between these 7 CpG sites. The link between gene methylation and gene expression was then assessed for SNP-associated CpG sites. From the 10 SNP-associated CpG sites analyzed, 4 were inversely correlated with gene expression levels while a trend toward a significant association was identified for 3 other sites (Table 6). Furthermore, mean methylation level of the group of 7 CpG sites located 1100–1402 bp upstream the TSS demonstrated inverse correlation with expression levels (*r* = −0.624; *P* = 0.02).

## 4. Discussion

Over the past years, differences in *DUSP1* gene expression have been reported in various types of cancers [15–21]. The current study reports underexpression of *DUSP1* in VAT of severely obese individuals with the MetS, thus extending the potential pathophysiological importance of this gene to obesity-related metabolic disease. Following *DUSP1* sequencing and identification of tSNPs, associations of *DUSP1* SNPs with CVD risk factors were identified, namely, HDL-C and fasting glucose levels. To provide potential explanation for these associations, further analyses were conducted on the three CVD risk factor-associated SNPs (rs881150, rs13184134, and rs7702178). Results presented here suggest that *DUSP1* SNPs modulate plasma glucose and HDL-C levels in severely obese patients and that this effect is potentially mediated through alterations of gene methylation and expression.

Our analysis of differentially expressed genes in VAT of severely obese individuals with and without MetS revealed a potential implication of the *DUSP1* gene in the interindividual variability observed in obesity-related metabolic complications. A gene dosage effect of *DUSP1* on IL-10 levels has been reported in response to lipopolysaccharide at the systemic level in mice and on macrophage stimulation [29]. This supports the hypothesis that differential expression of *DUSP1 *in VAT may have a physiological impact.

With genetic variations potentially having an impact on gene expression, assessment of SNPs was conducted and associations between selected SNPs and CVD risk factors (blood pressure, glucose, and lipid levels) were tested. Two SNPs (rs13184134 and rs7702178) were found to be associated with fasting glucose levels with a third one associated with HDL-C levels. DUSP1 is known to be involved in the regulation of innate immune function [11], inflammation [12, 13], and cellular response to oxidative stress [14]. Oxidative stress to endothelial cells and the subsequent decrease in glucose uptake and utilization have been shown to be responsible for insulin resistance [7]. Large-scale prospective studies have demonstrated that markers of inflammation predict incidence of T2D [30, 31]. Oxidative stress in adipocytes results in dysregulated expression of inflammation-related adipocytokines and is characterized by decreased secretion of adiponectin and increased proinflammatory adipocytokines [7]. In addition, an inverse relationship between elevated inflammation and low HDL-C has been demonstrated [32–34]. With obesity being characterized by low-grade inflammation [35], the impact of phenotype-associated SNPs identified here may be exerted through a modulation of DUSP1 antioxidative and anti-inflammatory functions.

Further analyses were performed to better understand the potential underlying mechanism for the difference in *DUSP1* gene expression levels and the associations identified for rs881150, rs13184134, and rs7702178. With SNPs having an impact on the binding of transcription factors with consequent impact on transcriptional activity [36] and gene expression levels [37], the first attempt assessed the impact of SNPs associated with cardiometabolic risk factors on transcription factor binding. The MatInspector program revealed that *DUSP1* promoter SNPs may potentially affect the binding of transcription factors and promoter transcriptional activity. With SNPs potentially having an impact on gene methylation leading to differences in gene expression [38, 39], the link between cardiometabolic risk factor-associated-SNPs, DNA methylation, and gene expression levels was assessed. The three SNPs associated with CVD risk factors (rs13184134, rs7702178, and rs881150) demonstrated associations with methylation levels of 10 different CpG sites. Most of the associations were found for rs881150 (702 bp upstream TSS) which associates with methylation levels of two CpG sites located in its vicinity (cg18121420 and cg02352687) and with 6 CpG sites located 1110–1402 bp upstream TSS. A trend toward an association with another CpG site (cg21121138) from this region was also identified. This suggests that the whole region is hypermethylated in carriers of the minor allele. Interestingly, it contains the glucocorticoid-responsive region shown to be involved in the anti-inflammatory effects of glucocorticoids (~1300 bp from TSS) [40, 41]. In regard to these results and the correlations found between *DUSP1* gene methylation and expression levels, it is tempting to speculate that associations identified here between *DUSP1* SNPs and cardiometabolic risk factors may be mediated through differences in CpG sites methylation and expression.

The current study has some limitations. Gene expression and DNA methylation analyses were conducted in a relatively small number of selected individuals from our larger cohort. However, individuals from this subset were free of metabolic diseases such as type 2 diabetes, cardiomyopathy, or endocrine disorders and were matched for age, BMI, and smoking status to patients with, on average, none of the recognized features of the MetS except high waist girth (MetS+ group, *N* = 7; MetS− group, *N* = 7) [10]. Gene expression and DNA methylation analyses were conducted on whole visceral adipose tissue samples. It is possible that other cell types found within adipose tissue such as endothelial cells, fibroblasts, or macrophages impact on tissue profiles. Differences observed here may be the result of differences in these cells or their proportion. Studies using isolated cell fractions and culture techniques will be required to elucidate this question. *DUSP1* resequencing and tSNPs selection were conducted in 25 individuals including the highly selected subset. Associations were thereafter tested in the whole cohort of 1906 individuals to identify SNPs explaining interindividual variability in obesity-related metabolic complications among severely obese patients. Looking at SNPs having a small impact on the phenotypes of interest, we deliberately chose to exclude testing the association of rare SNPs with obesity-related metabolic complications as our study had a limited power. Results presented here demonstrate an impact of *DUSP1* gene variations on obesity-related complications. Although the impact is of small effect size, these results might partially explain interindividual variability observed in metabolic complications among obese individuals and thus highlight a potential new MetS candidate gene. The present findings were obtained in a cohort of severely obese individuals. Because inflammation is found in severely obese individuals, the impact of *DUSP1* SNPs through altered regulation of the innate immune function may be subjected to different modulations. Thus, results from the present study cannot be extrapolated to individuals with less pronounced obesity levels or to the general population. It would be relevant to conduct similar studies in the general population or in a cohort of normal weight individuals showing low or moderate levels of inflammation.

In summary, the results obtained from the analysis of *DUSP1* promoter methylation and gene expression levels argue for an impact of *DUSP1* promoter SNPs on gene expression possibly mediated through alterations in specific CpG sites methylation levels. Combined with the associations identified here between SNPs and cardiometabolic risk factors, these results provide a potential novel contributor to the modulation of plasma glucose and HDL-C levels in obese patients. It would be interesting to identify pathways mediating the impact of *DUSP1* SNPs and to investigate how epigenetic differences at the human *DUSP1* locus relate to the large-interindividual variability observed in metabolic complications among obese individuals.

## Supplementary Material

Supplementary Table 1. Validation of microarray results by real-time PCR for the DUSP1 gene.
Supplementary Table 2. Potential impact of CVD-associated promoter SNPs on transcription factor binding sites.
Supplementary Table 3. List of CpG sites analyzed with corresponding localization.
Supplementary Figure 1. Schematic representation of sequence-derived SNPs at the DUSP1 locus (chromosome 5q34).

## Figures and Tables

**Table 1:** Subjects' characteristics.

	All^a^	MetS+^b^	MetS−^b^
Number of subjects (% male)	1906 (31.3%)	1549 (33.8%)	350 (20.9%)
Age (years)	43.1 ± 10.6	44.0 ± 10.6	38.9 ± 9.7
BMI (kg/m^2^)	51.6 ± 8.8	51.8 ± 8.9	50.9 ± 8.6
Waist girth (cm)	140.4 ± 17.6	141.5 ± 17.2 (100%)	135.4 ± 18.5 (100%)
Fasting glucose (mmol/L)	6.55 ± 2.35	6.87 ± 2.45 (74.2%)	5.13 ± 0.95 (10.9%)
Lipid profile			
Total-C (mmol/L)	4.68 ± 0.96	4.65 ± 0.99	4.79 ± 0.82
LDL-C (mmol/L)	2.66 ± 0.84	2.63 ± 0.85	2.79 ± 0.74
HDL-C (mmol/L)	1.23 ± 0.35	1.18 ± 0.32 (75.3%)	1.49 ± 0.36 (12.3%)
TG (mmol/L)	1.82 ± 1.07	1.96 ± 1.12 (67.7%)	1.17 ± 0.37 (2.9%)
Total-C/HDL-C	4.01 ± 1.28	4.16 ± 1.33	3.34 ± 0.75
Blood pressure			
SBP (mm Hg)	138.4 ± 17.1	139.5 ± 17.2 (88.7%)	133.8 ± 15.7 (58.9%)
DBP (mm Hg)	83.5 ± 11.5	83.7 ± 11.7 (77.9%)	82.7 ± 10.3 (47.7%)

Values are presented as mean ± SD. ^a^Seven individuals were not classified as MetS+ or MetS− due to missing data. ^b^Numbers into parenthesis represent the percentage of individuals meeting the corresponding MetS criterion. MetS+: metabolic syndrome affected individuals; BMI: body mass index; Total-C: total cholesterol; LDL-C: low-density lipoprotein cholesterol; HDL-C: high-density lipoprotein cholesterol; TG: triglycerides; SBP: systolic blood pressure; DBP: diastolic blood pressure.

**Table 2:** Characteristics of *DUSP1* sequence variations identified by direct sequencing in 25 severely obese individuals.

rs number	Other designation^a^	Region	MAF	HWE *P* value
rs322381	c. −1534C>T	Promoter	0.02	1.00
—	c. −1430G>A	Promoter	0.38	0.006
rs13184134	c. −1411C>T	Promoter	0.14	1.00
rs322382	c. −997A>G	Promoter	0.30	1.00
rs881150	c. −950A>T	Promoter	0.26	0.96
—	c. −648G>T	Promoter	0.02	1.00
rs2070996	c. −545C>T	Promoter	0.16	1.00
rs28372789	c. −333delA	Promoter	0.46	0.06
—	c. 106A>C; p.Asn36His	Exon 1	0.02	1.0
—	c. 120C>T; p.Ile40Ile	Exon 1	0.02	1.0
rs200878863	c. 158G>T; p.Arg53Leu	Exon 1	0.02	1.0
rs201026723	c. 180G>C; p.Met60Ile	Exon 1	0.04	1.0
rs34507926	c. 367+8C>G	Intron 1	0.04	1.0
rs35084382	c. 513+125A>G	Intron 2	0.02	1.0
rs7702178	c. 513+167A>G	Intron 2	0.16	1.0
rs2431663	c. 600C>A; p.Ile200Ile	Exon 3	0.02	1.0

^a^Reference sequences: NM_004417 and NP_004408. MAF: minor allele frequency; HWE: Hardy-Weinberg equilibrium.

**Table 3:** Genotype distribution of selected SNPs.

SNPs	Number of genotypes	Common HMZ	HTZ	Rare HMZ	MAF	HWE *P* value
rs13184134	1875	1288	519	68	0.17	0.10
rs322382	1857	928	780	149	0.29	0.44
rs881150	1851	1095	628	128	0.24	0.005
rs28372789	1851	522	939	390	0.46	0.44
rs201026723	1804	1747	57	0	0.02	1.00
rs7702178	1855	1264	530	61	0.18	0.56

SNP: single nucleotide polymorphism; HMZ: homozygotes; HTZ: heterozygotes; MAF: minor allele frequency; HWE: Hardy-Weinberg equilibrium.

**Table 4:** Genotype differences identified between fasting glucose, lipid profile, and *DUSP1* gene variations.

	rs13184134				rs7702178				rs881150			
Phenotypes	Means^a^			*P* values^b^	Means^a^			*P* values^b^	Means^a^			*P* values^b^
	Common HMZ	HTZ	Rare HMZ		Common HMZ	HTZ	Rare HMZ		Common HMZ	HTZ	Rare HMZ	
Fasting glucose (mM)	6.48 ± 2.20	6.62 ± 2.52	7.36 ± 3.23	**0.04**	6.51 ± 2.24	6.61 ± 2.49	7.26 ± 3.20	**0.01**	6.60 ± 2.35	6.46 ± 2.34	6.63 ± 2.46	0.99
Lipid profile												
Total-C (mM)	4.68 ± 0.95	4.67 ± 0.95	4.51 ± 1.00	0.67	4.67 ± 0.95	4.67 ± 0.96	4.49 ± 1.03	0.33	4.65 ± 0.95	4.72 ± 0.92	4.61 ± 1.10	0.81
LDL-C (mM)	2.66 ± 0.82	2.67 ± 0.84	2.51 ± 0.86	0.63	2.65 ± 0.82	2.66 ± 0.85	2.50 ± 0.88	0.22	2.63 ± 0.84	2.71 ± 0.79	2.62 ± 0.94	0.51
HDL-C (mM)	1.23 ± 0.37	1.24 ± 0.32	1.24 ± 0.29	0.39	1.23 ± 0.37	1.25 ± 0.33	1.25 ± 0.30	0.44	1.25 ± 0.35	1.23 ± 0.35	1.18 ± 0.34	**0.01**
TG (mM)	1.84 ± 1.16	1.77 ± 0.86	1.80 ± 0.85	0.95	1.84 ± 1.16	1.76 ± 0.86	1.79 ± 0.89	0.98	1.81 ± 1.07	1.80 ± 0.98	1.91 ± 1.48	0.93
Total-C/HDL-C	4.05 ± 1.34	3.98 ± 118	3.76 ± 0.96	0.32	4.04 ± 1.35	3.94 ± 1.18	3.73 ± 0.97	0.36	3.94 ± 1.17	4.10 ± 1.46	4.14 ± 1.32	0.07
Blood pressure												
SBP (mm Hg)	138.7 ± 17.0	137.6 ± 16.0	136.1 ± 18.7	0.46	138.5 ± 16.9	137.9 ± 16.1	134.4 ± 17.9	0.38	138.3 ± 17.2	138.7 ± 16.2	137.4 ± 17.9	0.65
DBP (mm Hg)	83.4 ± 11.3	83.6 ± 10.9	81.3 ± 11.1	0.42	83.4 ± 11.3	83.7 ± 11.0	80.5 ± 11.8	0.25	83.3 ± 11.3	83.8 ± 11.0	82.7 ± 11.9	0.70

Boldface values represent statistically significant results (*P* ≤ 0.05).

^a^Values presented (means ± SD) are untransformed and unadjusted.

^b^ *P* values obtained are adjusted for the effect of age, sex, and BMI.

HMZ: homozygote; HTZ: heterozygote; Total-C: total cholesterol; LDL-C: low-density lipoprotein cholesterol; HDL-C: high-density lipoprotein cholesterol; TG: triglycerides; SBP: systolic blood pressure; DBP: diastolic blood pressure.

**Table:** (a)

SNP	Localization	cg22473727	cg16957313	cg08452061	cg09493150	cg21121138	cg09799633	cg19537645	cg14968860
		Promoter	Promoter	Promoter	Promoter	Promoter	Promoter	Promoter	Promoter
rs13184134	Promoter	0.30	0.20	0.49	0.16	0.31	0.31	0.02^a^	0.77
rs881150	Promoter	0.34	0.28	0.06	0.04^a^	0.06	0.05^b^	0.01^b^	0.03^a^
rs7702178	Intron 2	0.06	0.07	0.16	0.04^a^	0.06	0.22	0.09	0.49

**Table:** (b)

SNP	Localization	cg23002268	cg25108022	cg02352687	cg18121420	cg04525476	cg11757894	cg08293091
		Promoter	Promoter	Promoter	Promoter	Promoter	Promoter	Intron 1
rs13184134	Promoter	0.74	0.82	0.61	0.53	0.91	0.67	0.03^a^
rs881150	Promoter	0.01^b,c^	0.006^b,c^	0.01^b^	0.02^b^	0.002^a,b,c^	0.06	0.78
rs7702178	Intron 2	0.95	0.96	0.62	0.83	0.83	0.87	0.06

Boldface values represent statistically significant results (*P* ≤ 0.05).

^a^Heterozygote genotype demonstrates higher methylation levels versus common homozygote genotype.

^b^Rare homozygote genotype demonstrates higher methylation levels versus common homozygote genotype.

^c^Rare homozygote genotype demonstrates higher methylation levels versus heterozygote genotype.

**Table 6:** Correlation of gene methylation with gene expression levels for SNPs-associated CpG sites.

CpG site ID	Localization	Correlation coefficient^a^	*P* value
cg09493150	Prom	−0.490	0.08
cg09799633	Prom	−**0.559**	**0.04**
cg19537645	Prom	−0.514	0.06
cg14968860	Prom	−**0.575**	**0.03**
cg23002268	Prom	−**0.535**	**0.05**
cg25108022	Prom	−0.480	0.08
cg02352687	Prom	−**0.579**	**0.03**
cg18121420	Prom	−0.392	0.17
cg04525476	Prom	−0.147	0.62
cg08293091	Ex1	−0.089	0.76

^a^Pearson's *r* correlation coefficient.

## References

[1] Mokdad AH, Ford ES, Bowman BA (2003). Prevalence of obesity, diabetes, and obesity-related health risk factors, 2001. *Journal of the American Medical Association*.

[2] Tirosh A, Shai I, Afek A (2011). Adolescent BMI trajectory and risk of diabetes versus coronary disease. *The New England Journal of Medicine*.

[3] Lafontan M, Girard J (2008). Impact of visceral adipose tissue on liver metabolism. Part I: heterogeneity of adipose tissue and functional properties of visceral adipose tissue. *Diabetes and Metabolism*.

[4] Mathieu P, Poirier P, Pibarot P, Lemieux I, Després J-P (2009). Visceral obesity the link among inflammation, hypertension, and cardiovascular disease. *Hypertension*.

[5] Cleeman JI (2001). Executive summary of the third report of the National Cholesterol Education Program (NCEP) expert panel on detection, evaluation, and treatment of high blood cholesterol in adults (adult treatment panel III). *Journal of the American Medical Association*.

[6] Karelis AD, St-Pierre DH, Conus F, Rabasa-Lhoret R, Poehlman ET (2004). Metabolic and body composition factors in subgroups of obesity: what do we know?. *Journal of Clinical Endocrinology and Metabolism*.

[7] Otani H (2011). Oxidative stress as pathogenesis of cardiovascular risk associated with metabolic syndrome. *Antioxidants and Redox Signaling*.

[8] Romeo GR, Lee J, Shoelson SE (2012). Metabolic syndrome, insulin resistance, and roles of inflammation—mechanisms and therapeutic targets. *Arteriosclerosis, Thrombosis, and Vascular Biology*.

[9] Teran-Garcia M, Bouchard C (2007). Genetics of the metabolic syndrome. *Applied Physiology, Nutrition, and Metabolism*.

[10] Bouchard L, Tchernof A, Deshaies Y (2007). ZFP36: a promising candidate gene for obesity-related metabolic complications identified by converging genomics. *Obesity Surgery*.

[11] Abraham SM, Clark AR (2006). Dual-specificity phosphatase 1: a critical regulator of innate immune responses. *Biochemical Society Transactions*.

[12] Kracht M, Saklatvala J (2002). Transcriptional and post-transcriptional control of gene expression in inflammation. *Cytokine*.

[13] Saklatvala J, Dean J, Clark A (2003). Control of the expression of inflammatory response genes. *Biochemical Society Symposium*.

[14] Yin Y, Terauchi Y, Solomon GG (1998). Involvement of p85 in p53-dependent apoptotic response to oxidative stress. *Nature*.

[15] Bang Y-J, Kwon JH, Kang SH, Kim JW, Yang YC (1998). Increased MAPK activity and MKP-1 overexpression in human gastric adenocarcinoma. *Biochemical and Biophysical Research Communications*.

[16] Vicent S, Garayoa M, López-Picazo JM (2004). Mitogen-activated protein kinase phosphatase-1 is overexpressed in non-small cell lung cancer and is an independent predictor of outcome in patients. *Clinical Cancer Research*.

[17] Wang H-Y, Cheng Z, Malbon CC (2003). Overexpression of mitogen-activated protein kinase phosphatases MKP1, MKP2 in human breast cancer. *Cancer Letters*.

[18] Calvisi DF, Pinna F, Meloni F (2008). Dual-specificity phosphatase 1 ubiquitination in extracellular signal-regulated kinase-mediated control of growth in human hepatocellular carcinoma. *Cancer Research*.

[19] Rauhala HE, Porkka KP, Tolonen TT, Martikainen PM, Tammela TLJ, Visakorpi T (2005). Dual-specificity phosphatase 1 and serum/glucocorticoid-regulated kinase are downregulated in prostate cancer. *International Journal of Cancer*.

[20] Shimada K, Nakamura M, Ishida E (2007). c-Jun NH2 terminal kinase activation and decreased expression of mitogen-activated protein kinase phosphatase-1 play important roles in invasion and angiogenesis of urothelial carcinomas. *American Journal of Pathology*.

[21] Tsujita E, Taketomi A, Gion T (2005). Suppressed MKP-1 is an independent predictor of outcome in patients with hepatocellular carcinoma. *Oncology*.

[22] Marceau P, Hould F-S, Simard S (1998). Biliopancreatic diversion with duodenal switch. *World Journal of Surgery*.

[23] Robitaille J, Després J-P, Pérusse L, Vohl M-C (2003). The PPAR-gamma P12A polymorphism modulates the relationship between dietary fat intake and components of the metabolic syndrome: results from the Québec Family Study. *Clinical Genetics*.

[24] Barrett JC, Fry B, Maller J, Daly MJ (2005). Haploview: analysis and visualization of LD and haplotype maps. *Bioinformatics*.

[25] De Bakker PIW, Yelensky R, Pe’Er I, Gabriel SB, Daly MJ, Altshuler D (2005). Efficiency and power in genetic association studies. *Nature Genetics*.

[26] Cartharius K, Frech K, Grote K (2005). MatInspector and beyond: promoter analysis based on transcription factor binding sites. *Bioinformatics*.

[27] Quandt K, Frech K, Karas H, Wingender E, Werner T (1995). Matlnd and Matlnspector: new fast and versatile tools for detection of consensus matches in nucleotide sequence data. *Nucleic Acids Research*.

[28] Reese MG, Eeckman FH, Kulp D, Haussler D (1997). Improved splice site detection in Genie. *Journal of Computational Biology*.

[29] Chi H, Barry SP, Roth RJ (2006). Dynamic regulation of pro- and anti-inflammatory cytokines by MAPK phosphatase 1 (MKP-1) in innate immune responses. *Proceedings of the National Academy of Sciences of the United States of America*.

[30] Duncan BB, Schmidt MI, Pankow JS (2003). Low-grade systemic inflammation and the development of type 2 diabetes: the atherosclerosis risk in communities study. *Diabetes*.

[31] Pradhan AD, Manson JE, Rifai N, Buring JE, Ridker PM (2001). C-reactive protein, interleukin 6, and risk of developing type 2 diabetes mellitus. *Journal of the American Medical Association*.

[32] Barter P, McPherson YR, Song K (2007). Serum insulin and inflammatory markers in overweight individuals with and without dyslipidemia. *Journal of Clinical Endocrinology and Metabolism*.

[33] Clarke R, Emberson JR, Breeze E (2008). Biomarkers of inflammation predict both vascular and non-vascular mortality in older men. *European Heart Journal*.

[34] Pineda J, Marín F, Marco P (2009). Premature coronary artery disease in young (age < 45) subjects: interactions of lipid profile, thrombophilic and haemostatic markers. *International Journal of Cardiology*.

[35] Ross R (1999). Atherosclerosis—an inflammatory disease. *The New England Journal of Medicine*.

[36] Ponomarenko JV, Orlova GV, Merkulova TI (2002). rSNP_Guide: an integrated database-tools system for studying SNPs and site-directed mutations in transcription factor binding sites. *Human Mutation*.

[37] Grundberg E, Small KS, Hedman AK (2012). Mapping cis- and trans-regulatory effects across multiple tissues in twins. *Nature Genetics*.

[38] Bell JT, Pai AA, Pickrell JK (2011). DNA methylation patterns associate with genetic and gene expression variation in HapMap cell lines. *Genome Biology*.

[39] Drong AW, Nicholson G, Hedman AK (2013). The presence of methylation quantitative trait Loci indicates a direct genetic influence on the level of DNA methylation in adipose tissue. *PLoS One*.

[40] Johnasson-Haque K, Palanichamy E, Okret S (2008). Stimulation of MAPK-phosphatase 1 gene expression by glucocorticoids occurs through a tethering mechanism involving C/EBP. *Journal of Molecular Endocrinology*.

[41] Tchen CR, Martins JRS, Paktiawal N, Perelli R, Saklatvala J, Clark AR (2010). Glucocorticoid regulation of mouse and human dual specificity phosphatase 1 (*DUSP1*) genes: unusual cis-acting elements and unexpected evolutionary divergence. *Journal of Biological Chemistry*.

