# Supplementary material for: DUSP1 Gene Polymorphisms Are Associated with Obesity-Related Metabolic Complications among Severely Obese Patients and Impact on Gene Methylation and Expression
Source: Int J Genomics. 2013 Aug 6;2013:609748. doi: 10.1155/2013/609748 (PMC3748404; doi:10.1155/2013/609748)
Supplement: Supplementary file 1 — Supplementary Table 1. Validation of microarray results by real-time PCR for the DUSP1 gene. Supplementary Table 2. Potential impact of CVD-associated promoter SNPs on transcription factor binding sites. Supplementary Table 3. List of CpG sites analyzed with corresponding localization. Supplementary Figure 1. Schematic representation of sequence-derived SNPs at the DUSP1 locus (chromosome 5q34). [file 609748.f1.doc]

**Supplementary Table 1. Validation of microarray results by RT-PCR for the *DUSP1* gene.**

|  |  | Microarray | |  | RT-PCR | |  | Correlation between microarray and RT-PCR results |
| --- | --- | --- | --- | --- | --- | --- | --- | --- |
| MetS group |  | Mean expression | MFED (MetS+/MetS-) |  | Mean expression (ΔC_T_) | MFED (2^-ΔΔCT^) |  |
| MetS+ |  | 8966 | -3.77 (p = 0.02) |  | 1.175 | -6.02 (p = 0.04) |  | r = 0.984 (p < 0.0001) |
| MetS- |  | 33 762 |  |  | 3.764 |  |  |  |

Negative MFED values mean that the gene is underexpressed in the MetS+ group.

Abbreviations: MetS, metabolic syndrome; MetS+, with the MetS; MetS-, without the MetS; MFED, mean fold expression difference; RT-PCR, real-time PCR.

**Supplementary Table 2.** Potential impact of CVD-associated promoter SNPs on TFBS

| **SNP** | **TFBS** | **Strand** | **Matrix sim (WT/var)** | **Potential impact** |
| --- | --- | --- | --- | --- |
| rs13184134 | PBX1/MEIS1 heterodimer | (-) | 0.797/0.790 | --- |
|  | Nuclear factor 1 (CTF1) | (+) | 0.855/0.860 | --- |
|  | Nuclear factor 1 (CTF1) | (-) | ND/0.820 | Creation of a TFBS |
|  | Sp4 transcription factor | (-) | 0.873/ND | Disruption of a TFBS |
|  | Krueppel-like factor 7 | (-) | 0.911/ND | Disruption of a TFBS |
|  | REX1 transcription factor; zinc finger protein 42 | (-) | ND/0.900 | Creation of a TFBS |
|  | Se-Cys tRNA gene transcription activating factor | (+) | 0.777/0.798 | --- |
|  | THAP domain containing, apoptosis associated factor | (-) | ND/0.917 | Creation of a TFBS |
|  |  |  |  |  |
| rs881150 | Thing1/E47 heterodimer | (-) | ND/0.937 | Creation of a TFBS |
|  | B-cell CLL/lymphoma 6, member B (BCL6B) | (-) | 0.895/ND | Disruption of a TFBS |
|  | Mammalian transcriptional repressor RBP-Jkappa/CBF1 | (+) | 0.941/ND | Disruption of a TFBS |
|  | AREB6 (Atp1a1 regulatory element binding factor 6) | (-) | 0.988/ND | Disruption of a TFBS |

Abbreviations: Matrix sim, matrix similarity; ND, not detected; SNP, Single Nucleotide Polymorphism; TFBS, Transcription Factor Binding Site; Var, variant allele; WT, wild type allele.

**Supplementary Table 3.** List of CpG sites analyzed with corresponding localization

| **CpG site ID** | **Position** | **Localization** |
| --- | --- | --- |
| cg22473727 | chr5:172199667 | Promoter |
| cg16957313 | chr5:172199661 | Promoter |
| cg08452061 | chr5:172199642 | Promoter |
| cg09493150 | chr5:172199605 | Promoter |
| cg21121138 | chr5:172199485 | Promoter |
| cg09799633 | chr5:172199460 | Promoter |
| cg14450231 | chr5:172199425 | Promoter |
| cg19537645 | chr5:172199418 | Promoter |
| cg14968860 | chr5:172199365 | Promoter |
| cg23002268 | chr5:172199318 | Promoter |
| cg25108022 | chr5:172199313 | Promoter |
| cg12333707 | chr5:172199303 | Promoter |
| cg06819445 | chr5:172199278 | Promoter |
| cg23162310 | chr5:172199255 | Promoter |
| cg10837843 | chr5:172199244 | Promoter |
| cg10672508 | chr5:172199157 | Promoter |
| cg20534525 | chr5:172199132 | Promoter |
| cg02352687 | chr5:172199101 | Promoter |
| cg18121420 | chr5:172199013 | Promoter |
| cg15414042 | chr5:172198979 | Promoter |
| cg05612977 | chr5:172198876 | Promoter |
| cg04060275 | chr5:172198791 | Promoter |
| cg12884779 | chr5:172198737 | Promoter |
| cg04525476 | chr5:172198714 | Promoter |
| cg11757894 | chr5:172198326 | Promoter |
| cg19801141 | chr5:172198162 | Exon 1 |
| cg17946497 | chr5:172198141 | Exon 1 |
| cg22229034 | chr5:172198031 | Exon 1 |
| cg17429424 | chr5:172197911 | Exon 1 |
| cg26095194 | chr5:172197489 | Intron 1 |
| cg08293091 | chr5:172197350 | Intron 1 |
| cg15890707 | chr5:172197278 | Exon 2 |
| cg17378966 | chr5:172196746 | Exon 3 |
| cg00593243 | chr5:172196153 | Intron 3 |
| cg07018389 | chr5:172195678 | Exon 4 |
| cg02029908 | chr5:172195602 | Exon 4 |


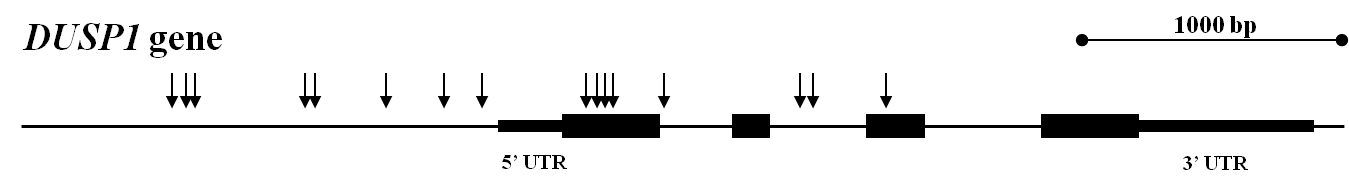


**Supplementary Figure 1.** Schematic representation of sequence-derived SNPs at the *DUSP1* locus (chromosome 5q34). SNPs identified during the sequencing of the promoter (~1500 bp), exons and intronic flanking regions of the *DUSP1* gene are indicated by arrows.
